# Mitochondrial Fus1/Tusc2 and cellular Ca2^+^ homeostasis: tumor suppressor, anti-inflammatory and anti-aging implications

**DOI:** 10.1038/s41417-022-00434-9

**Published:** 2022-02-18

**Authors:** Roman Uzhachenko, Akiko Shimamoto, Sanika S. Chirwa, Sergey V. Ivanov, Alla V. Ivanova, Anil Shanker

**Affiliations:** 1 Department of Biochemistry, Cancer Biology, Neuroscience and Pharmacology, School of Medicine, Meharry Medical College, Nashville, TN USA; 2 School of Graduate Studies and Research, Meharry Medical College, Nashville, TN USA; 3 Host-Tumor Interactions Research Program, Vanderbilt-Ingram Cancer Center, Vanderbilt University, Nashville, TN USA; 4 Vanderbilt Institute for Infection, Immunology and Inflammation, Vanderbilt University, Nashville, TN USA; 5 Vanderbilt Memory and Alzheimer’s Center, Vanderbilt University, Nashville, TN USA; 6 Department of Cell and Developmental Biology, Vanderbilt University, Nashville, TN USA

**Keywords:** Cancer, Cancer prevention

## Abstract

*FUS1/TUSC2* (*FUS*ion*1*/TUmor *S*uppressor *C*andidate *2*) is a tumor suppressor gene (TSG) originally described as a member of the TSG cluster from human 3p21.3 chromosomal region frequently deleted in lung cancer. Its role as a TSG in lung, breast, bone, and other cancers was demonstrated by several groups, but molecular mechanisms of its activities are starting to unveil lately. They suggest that Fus1-dependent mechanisms are relevant in etiologies of diseases beyond cancer, such as chronic inflammation, bacterial and viral infections, premature aging, and geriatric diseases. Here, we revisit the discovery of *FUS1* gene in the context of tumor initiation and progression, and review 20 years of research into FUS1 functions and its molecular, structural, and biological aspects that have led to its use in clinical trials and gene therapy. We present a data-driven view on how interactions of Fus1 with the mitochondrial Ca^2+^ (mitoCa^2+^) transport machinery maintain cellular Ca^2+^ homeostasis and control cell apoptosis and senescence. This Fus1-mediated cellular homeostasis is at the crux of tumor suppressor, anti-inflammatory and anti-aging activities.

## Introduction

Execution of cellular programs including proliferation, differentiation, apoptosis, senescence is based on fine-tuned signal transduction cascades. Growth receptor signaling (such as receptor tyrosine kinases or RTK) should be tightly controlled at the spatial and temporal levels to execute appropriate cell decisions. Uncontrolled RTK signaling (via EGFR, HER2/neu, or VEGFR) could be oncogenic and conducive to a variety of other pathologies [1, 2]. Indeed, tumor suppressors integrate various signaling networks pivotal for tissue homeostasis [1, 2]. They control DNA repair (e.g., BRCA1, MSH2), cell cycle progression (RB1, CDKN2A, TGFB1), angiogenesis (VEGF, ANGPTL4), apoptosis (TP53, BCL2), and cell adhesion (CADM1, FAT, CDH) [1–8]. A separate class of tumor suppressors is miRNAs, small hairpin RNAs regulating gene expression (miR-7, miR-29, miR-145) [5, 6, 9] and https://bioinfo.uth.edu/TSGene/. Loss of TSG functions in malignant cells occurs at the DNA, transcriptional and post-transcriptional levels and may be achieved via different mechanisms. According to the Knudson’s two-hit paradigm (stemming from clinical observations on *Rb* deficiency), first hit comes from the loss of heterozygosity (LOH) due to deletion or loss-of-function mutation in one of two TSG alleles that can be inherited as a recessive mutation. Second hit causes a biallelic mutation eliminating the function of the remaining TSG allele. This transforms the cell towards malignancy. The effect of haploinsufficiency, when mutation of a single gene copy is sufficient for malignant transformation, has been described for some TSGs (CDKN1B, TP53, NF1, PTEN) [7, 8]. Later, importance of epigenetic silencing via promoter hypermethylation or histone modification was also recognized [7, 8]. At the protein level, TSG products can be functionally inactivated via proteasomal degradation (MDM2/p53 axis) or through altered cellular compartmentalization (mislocalization of SMAD4 in cytosol vs. nucleus) [5]. Transcriptional silencing of TSGs in tumors has also been reported (TWIST/CDH1 repression axis) [5].

In this review, tracing the history of the tumor suppressor *Fus1/Tusc2* studies “from bench to bedside”, we discuss what is known of its role in tumor development, inflammation, and aging. Initially characterized as one of several genes belonging to potential 3p21.3 TSG cluster [10–12], recently FUS1 gene enters clinical trials as a gene-based therapy in the form of plasmid DNA encapsulated in cation lipid nanopartciles (REQORSA) in combination with other drugs for patients with non-small cell lung cancer (NSCLC) [13].

Reported ability of Fus1/Tusc2 to inhibit receptor (EGFR, PDGFR, c-Kit) and non-receptor (c-Abl, Akt) tyrosine kinases as well as promote apoptosis [14] makes Fus1/Tusc2 a promising adjuvant for successful anti-tumor chemotherapy (https://clinicaltrials.gov/ct2/show/NCT01455389, https://clinicaltrials.gov/ct2/show/NCT04486833). By affecting the listed above upstream pathways, Fus1/Tusc2 can potentailly prevent cancer cell evolution towards drug-resistant variants. During the last years, ongoing revolution in immunotherapy including series of Abs targeting immune checkpoint (IC) molecules altered therapeutic landscape in tumor treatment and significantly improved therapeutic outcomes [15–17]. However, broad spectrum of immune evasion mechanisms let cancer cells escape from immune surveillance stimulated by using IC inhibitors in clinics (e.g., expression of alternative IC molecules such as TIM-3 after anti-PD-1 therapy) [18, 19]. This issue was suggested to be solved by using REQORSA as Fus1/Tusc2-based genetic therapy in mouse model of lung cancer reduced expression of array of IC molecules (TIM-3, CTLA-4, PD-1) concomitant with anti-tumor response [20].

Noteworthy, studies on mice with the Fus1 loss extended its biological activity beyond tumorigenesis to aging, inflammation, infections, and geriartic conditions [21–23]. Fus1 was demonstrated to be the novel regulator of mitochondrial Ca^2+^ (miCa^2+^) transport [24, 25, 23]. Fine-tuning of Ca^2+^ signaling by Fus1/Tusc2 may control cellular senescence and its disturbance may contribute to various pathologies.

## *Fus1/Tusc2* gene discovery and function

The pioneering concept describing the importance of loss of chromosomal segments responsible for tumor suppression was put forward by Theodor Boveri in 1914 [26]. In the 1980s, chromosomal abnormalities identified in small cell lung carcinoma (SCLC) demonstrated that SCLC cell lines and tumor biopsies shared the deletion of 3p14-23 region [27, 28]. Further, this chromosomal segment was narrowed down to 3p [21–23, 29] and 3p21 [30] as two most common overlapping deletions in SCLC and other lung cancers. In parallel, it was established that the 3p deletions followed the LOH pattern and matched the Knudson’s two-hit paradigm [31, 32]. The localization of TSGs in the 3p21 region was reinforced by detection of deletions in this area in other solid (e.g., breast, cervix, and renal carcinoma) and hematopoietic (chronic myeloid leukemia) tumors [33–41]. Few research groups used a microcell fusion technique to transfer 3d chromosome into tumor cells. Such hybrid tumor cells displayed signs of senescence, growth arrest, and decreased tumorigenicity in athymic nude mice. This demonstrated a tumor suppressor potential for the entire chromosome 3 [42] and regions 3p21 [43] and 3p21.3 [44].

To perform detailed mapping of the 3p21 region, initial analysis of growth suppression of human-mouse tumor hybrid cells was done and resulted in the isolation of a subclone HA [3] BB9F carrying a 2-Mb fragment of human 3p21-22 chromosome with tumor suppressor function [43]. Further, this region was narrowed down to the 3p21.2-21.3 area using specific chromosomal markers [45]. A long-range physical map spanning about 1.8-Mb DNA over the deleted region helped to construct a 700-kb clone. This library (23 cosmids and one PI phage) included a genetically defined 370-kb segment containing lung cancer TSGs from the 3p21.3 fragment [40]. This led to the identification of a 220 kb segment deleted in primary breast cancer. By overlapping chromosomal fragments deleted in SCLC cells, the TSGs area was narrowed down to the minimal 120 kb deletion nested within three small homozygous deletions in the 3p21.3 segment (3p21.3 C or LUCA, 3p21.3 or CER1, and 3p21.3 T or AP20). Within this 120 kb deletion were identified eight genes: *HYAL2*, *FUS1(TUSC2)*, *RASSF1*, *BLU/ZMYND10*, *NPR2L*, *101F6*, *PL6*, and *CACNA2D2* [11]. The original name of *TUSC2*, *FUS1*, was introduced based on its position at the junction (FUSion) of cosmids LUCA12 and LUCA13. *FUS1* remains to be commonly used even though it is sometimes confused with an unrelated gene, *FUS* [10].

Shortly after gene identification, it was demonstrated that overexpression of the *FUS1/TUSC2* transgene in *FUS1/TUSC2*-deficient lung cancer cells suppressed proliferation, blocked G1/S or G2/M transition, and increased doubling time implying a tumor suppressor role for FUS1/TUSC2 [46, 47]. Likewise, intratumoral adenoviral delivery of the *Fus1/Tusc2* transgene suppressed tumor growth and lung metastases in mice [46].

For further insights into the biological role of FUS1, *Fus1/Tusc2*^*−/−*^ mice were developed. These animals showed increased frequencies of lupus-like autoimmune conditions (vasculitis, glomerulonephritis, anemia, circulating autoantibodies) and spontaneous vascular tumors [22], confirming tumor suppressor properties of Fus1. Moreover, these mice exhibited increased susceptibility to irradiation [48, 49], enhanced response to *A. baumanii* infection [21], premature aging [23], hearing loss [50], and olfactory and spatial memory impairments [51]. These pleiotropic effects of Fus1/Tusc2 loss expanded its role beyond tumor suppression activities. Also, these data suggested that Fus1/Tusc2-dependent therapeutic approaches could alleviate/treat different diseases associated with mitochondrial dysfunction, which is linked to inflammation, infection, metabolic imbalance, and aging.

## *Fus1/Tusc2* localization, mutations, and expression in normal and tumor tissues

**Fig. 1:**
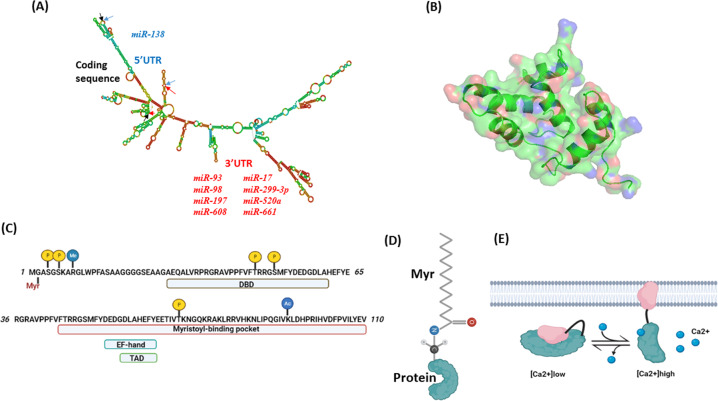
Structural properties of Fus1/Tusc2 gene and protein. **A** Secondary structure of human mRNA encoding FUS1/TUSC2 protein. Modeling of secondary mRNA structure was performed using RNAFold Webserver [141]. Arrows of different colors indicate upstream and downstream borders of 5’ UTR (blue), coding sequence (black), and 3’ UTR (red). miRNAs binding to 5’ and 3’ UTRs are shown in blue and red, respectively. The mRNA sequence has been retrieved from NCBI (NM_007275.3). **B** Predicted tertiary structure of Fus1/Tusc2 protein. Modeling was performed using the SWISS-MODEL [142] server and protein sequence obtained from NCBI (NP_009206.1). Human recoverin (acc. #. 2D8N) has been used as a template. The 3D protein structure has been visualized using PyMOL software (The PyMOL Molecular Graphics System, Version 2.0 Schrödinger, LLC; https://pymol.org/2/#page-top). **C** Computationally and experimentally predicted protein motifs and posttranslational modifications in the amino acid structure of Fus1/Tusc2 including myristoylation site (Myr), DNA binding domain (DBD), Ca^2+^ binding motif (EF-hand), transactivation domain (TAD), and myristoyl-binding pocket. Phosphorylation sites are labeled with yellow circles, methylation and acetylation sites are labeled with blue circles. **D** Schematic depiction of myristoylated protein with the myristoyl tail conjugated to the N-end of the protein. **E** Schematic outline of Ca^2+^/myristoyl switch protein activity. At low Ca^2+^ levels, myristoyl tail resides inside the hydrophobic pocket, and protein remains in inactive state. At high Ca^2+^ levels, Ca^2+^ binding to the EF-hand motif unlocks the myristoyl tail thereby anchoring polypeptide to the membrane. Protein tethering to membranes results in protein activation and interaction with the binding partners.

The *FUS1/TUSC2* gene is located in the centromeric segment of 3p21.3 (chromosome 9 in mice) encoding a small (110 aa) protein without recognizable domains [10, 12]. The *TUSC2/FUS1* gene is 3.3 kb long and contains three exons, which encode a 1691 bp mRNA with 5’UTR (untranslated region) spanning positions 1–147 and 3’UTR spanning positions 481–169 [10] (Fig. 1A).

At the beginning of 3p21.3 cluster analysis, it was expected that TSGs from this region would possess cancer-associated spectrum mutations. Surprisingly, extensive studies found little or no mutations in the genes from 3p21.3 cluster, and in the *FUS1/TUSC2* gene, except for a few lung cancer cell lines with gene deletions and stop mutations. Overall, the mutational rate in this area did not exceed 5% [10]. The identified stop mutation (28 bp deletion at the 3’ terminus of the *FUS1/TUSC2* exon 2) resulted in the expression of nonfunctional C-terminal truncation at position 82 [47].

In normal tissues*, FUS1/TUSC2* is ubiquitously expressed with the highest levels in all brain regions, followed by blood vessels, stomach, esophagus, colon, adrenal, pituitary, and thyroid glands, skeletal muscle, kidney, spleen, lung, testis, and fallopian tubes (https://gtexportal.org/home/gene/TUSC2).

*FUS1/TUSC2* mRNA was detected in lung and sarcoma cancer cell lines and sarcoma tissues, but no protein expression was detected in SCLC or non-small cell lung carcinoma (NSCLC) cells [2, 47, 52, 53]. Low or no protein expression was explained by its reduced half-life in tumor cells due to the loss of myristoylation, a post-translational modification that prevents FUS1/TUSC2 from proteasome-mediated degradation [53]. Detailed information on systemic, cellular and molecular manifestations of Fus1 decrease/loss/increase in normal and tumor tissues is presented in Table 1.

**Table 1:** Systemic, cellular and molecular manifestations of Fus1 decrease/loss/increase in normal and tumor tissues.

Type of tumor or normal tissue	Systemic and molecular effects of Fus1 loss/overexpression	Reference
3p21.3-deficient lung cancer cells H1299 and A549. (Fus1 is lost as the part of 3p21.3 deletion)	Overexpression of FUS1/TUSC2 transgene suppresses proliferation, blocks G1/S or G2/M transition, and increases doubling time Intratumoral adenoviral delivery of the FUS1/TUSC2 transgene suppressed growth of tumor xenografts and inhibited experimental lung metastases in nu/nu mice	[45, 46]
Human white blood cells, human keratinocyte cell line HaCaT, human bronchial epithelial cell line BEAS-2B, human breast cancer cell lines MDA-MB231, MB468 and MT-1, a human glioblastoma cell line U87, and a mouse breast cancer cell line 4T1. All cancer cells have lower (TUSC2 expression is lower in all cancer cells than in all normal cells in the study)	Increase of Fus1/Tusc2 mRNA expression after sequestration of miRNAs by TUSC2P inhibits cell proliferation, survival, migration, invasion, colony formation and stimulates tumor cell death	[61]
Human NSCLC cell lines A549, H1299, H358, H226, H322, H460, normal human lung fibroblast cell line WI-38. (TUSC2 expression is lower in all cancer cells than in a normal cell line)	Myristoylation-deficient FUS1/TUSC2 loses its abilities to induce apoptosis and suppress tumor cell proliferation in vitro and promotes tumor growth and metastases in vivo	[53]
Murine osteoclasts from bone marrow. Normal Tusc2 levels.	Overexpression of Tusc2 positively regulates osteoclast differentiation induced by RANKL. Tusc2 induces activation of Ca^2+^-dependent RANKL-mediated NF-κB and CaMKIV/CREB signaling cascades.	[71]
Human NSCLC cell lines A549 and H1299, normal human lung fibroblast 32D P210 cells. (TUSC2 expression is lower in all cancer cells than in a normal cell line)	Deletion of 83-110 aa at the FUS1/TUSC2 C-terminus leads to the loss of its ability to inhibit tyrosine kinase c-Abl	[98]
Human NSCLC cell lines H1299, H460, A549, H322 and normal human bronchial epithelial cells HBEC. (TUSC2 expression is lower in all cancer cells than in a normal cell line)	Co-expression of *FUS1/TUSC2* and *p53* synergistically increased apoptosis associated with down-regulation of MDM2 and activation of Apaf/caspase-3	[113]
Anaplastic thyroid cancer cell line 8505 C and papillary thyroid cancer cell line TPC-1 (Fus1 level is decreased)	Overexpression of *FUS1/TUSC2* increases levels of Smac/Diablo, suppressor of IAPs blocking caspase- and cytochrome *c-*mediated apoptosis	[115]
Cell-free (Protein Chip array and SELDI-TOF mass spectrometry)	Direct interaction between PDZ domains of FUS1/TUSC2 and Apaf	[116]
*Fus1 KO* mouse model (Fus1 is deleted in all tissues)	Increased frequencies of lupus-like autoimmune conditions (vasculitis, glomerulonephritis, anemia, circulating autoantibodies) and spontaneous vascular tumors, defective NK cell maturation in Fus1 KO mice completely rescued by in vivo injections of IL-15 expressing plasmid. Increased susceptibility to irradiation, enhanced response to *A. baumanii* infection, premature aging, hearing loss, and olfactory and spatial memory impairments.	[21–23, 48–50]
Gastrointestinal epithelial cells from irradiated *Fus1 KO* mice (Fus1 is deleted)	After in vivo irradiation, epithelial cells demonstrated accelerated cell cycle arrest, aberrant mitosis, lack of proper DNA repair (mitotic catastrophe), early activation of p53, inadequate cellular antioxidant defenses, defective redox homeostasism and death of gastrointestinal crypt cells. IR sensitivity in Fus1 KO cells could be alleviated by antioxidant treatment with Pyridoxamine.	[48, 49]
Activated mouse CD4^+^ T cells (Fus1 is deleted), human tumor cells (Fus1 is silenced)	Deletion or silencing of *Fus1/Tusc2* accelerates cell proliferation	[24, 129]
*Fus1 KO* CD4^+^ T cells, mouse embryonic fibroblasts, kidney epithelial cells (Fus1 is deleted)	Loss of Fus1 altered Ca^2+^ signaling including mitochondrial Ca^2+^ accumulation during cytosolic Ca^2+^ rises, which led to hyperactivation of basal NFAT/NFkB and decreased NFAT/NFkB activation during Ca^2+^ elevations induced by cell stimulation	[24, 25, 23]
Lung tissues and BALF cells from Fus1 KO mice infected with A. Baumanii (Fus1 is deleted)	Early recruitment of lymphocytes to infection site, early activation of anti-bacterial pathways, (PI3K/Akt/mTOR pathways activation, PTEN downregulation), increased mitochondrial membrane potential and UCP2 (UnCoupled Protein 2) expression	[21]
Peritoneal granulocytes from *Fus1/Tusc2 KO* mice (Fus1 is deleted)	After intraperitoneal injection of asbestos, infiltrating cells demonstrate signatures of enhanced genotoxic stress (elevated γH2AX, DNA damage response molecule, and phosphorylated pro-inflammatory NFκB and ERK1/2)	[70]
Gastrointestinal epithelial cells from *Fus1/Tusc2 KO* mice (*Fus1* is deleted)	After in vivo irradiation, epithelial cells demonstrated accelerated cell cycle arrest, aberrant mitosis, lack of proper DNA repair (mitotic catastrophe), early activation of p53, and death of gastrointestinal crypt cells	[49]
Head-and-neck cancer cells JHU012 (Fus1 is decreased), splenocytes, cochlear cells, epithelial cells from *Fus1 KO* mice (Fus1 is deleted)	Increased ROS production, up-regulation of antioxidant defense proteins (Prdx1) at steady-state, age-dependent decrease in the expression of Sod2 and Prdx1	[24, 50, 70]
*P*rimary mouse embryonic fibroblasts and immortalized mouse kidney epithelial cells from *Fus1 KO* mice (Fus1 is deleted)	Defects in respiration (significantly decreased maximal mitochondrial respiration and respiratory reserve capacity)	[23]
Cochlear cells from *Fus1 KO* mice (Fus1 is deleted)	Pathological alterations in antioxidant (AO) and nutrient and energy sensing pathways (mTOR and PTEN/AKT) and down-regulation of PINK1, a sensor of mitochondrial quality control occur in cochleae of young Fus1 KO mice before major hearing loss. Short-term anti-oxidant treatment corrected these pathological molecular changes and delayed hearing loss.	[50]

## *FUS1/TUSC2* gene regulation in normal and tumor tissues

Besides the post-translational modification affecting *FUS1/TUSC2* expression due to the loss of myristoylation, its expression can also be regulated at the translational level. At the 5’UTR, the *FUS1/TUSC2* mRNA contains two alternative highly conserved open reading frames (uORF1 and uORF2) and a secondary structure, which can suppress ribosomal scanning during translation. The 3’UTR also displays a negative regulatory activity mediated via miRNAs and regulation of mRNA stability [54]. Thus, miR-93, miR-98, and miR-197 target 3’UTR and down-regulate expression of *FUS1/TUSC2* mRNA (Fig. 1A, Table 2). Elevated miR-93 and miR-197 expression correlated with reduced *FUS1/TUSC2* expression in NSCLC tumors [55]. Other miRNAs suppressing *FUS1/TUSC2* mRNA expression include miR-663 in ovarian cancer [56], miR-19a in lung cancer [57], miR-378 in mesenchymal stem cells [58], and miR-584 in thyroid cancer [59]. In triple-negative breast cancer cells, miR-138 binds to the 5′-UTR site on *FUS1/TUSC2* mRNA containing translation initiation region and interferes with its translation [60]. An additional layer regulating *FUS1/TUSC2* mRNA expression includes two *FUS1/TUSC2* pseudogenes (TUSC2P) on chromosomes X and Y. Their RNAs have the region identical to the 3’UTR region of *FUS1/TUSC2* mRNA. *Tusc2P* mRNA is complimentary to several miRNAs: miR-17, miR-93, miR-299-3p, miR-520a, miR-608, and miR-661 (Fig. 1A). Their binding to *FUS1/TUSC2* RNA sequesters miRNAs from interacting with the 3’UTR of *FUS1/TUSC2* mRNA. As a result, *FUS1/TUSC2* mRNA expression is increased, leading to inhibited cell proliferation, survival, migration, invasion, colony formation as well as increased tumor cell death [61]. Potentially, miRNAs or proteins involved in the ribosome scanning of 5’-UTR can alter *FUS1/TUSC2* mRNA stability or its translation during progression from normal bronchial epithelium to malignancy [54].

**Table 2:** A list of miRNA molecules regulating Fus1/Tusc2 levels in various cancers.

Name	UTR on mRNA	Expression, tissue	Reference
miR-93 miR-98	3’	Expressed at higher levels in SCLC lines compared to NSCLC lines and immortalized human bronchial epithelial cells (HBECs); miR-93 binds to TUSC2P mRNA (see below)	[55, 61]
miR-663	3’	Ovarian cancer	[56]
miR-197	3’	Expressed at higher levels in both SCLC and NSCLC compared to HBECs	[55]
miR-19a	3’	Lung cancer	[57]
miR-378	3’	Mesenchymal stem cells	[58]
miR-584	3’	Thyroid cancer	[59]
miR-138	5’	Triple-negative breast cancer	[60]
miR-17 miR-93 miR-299-3p miR-520a miR-608 miR-661	3’	Binds to TUSC2P mRNA expressed at high levels in normal cells (human white blood cells, human keratinocyte cell line HaCaT, and human bronchial epithelial cell line BEAS-2B). TUSC2P mRNA expressed at low levels in human breast cancer cell lines (MDA-MB231, MB468 and MT-1), a human glioblastoma cell line U87, and a mouse breast cancer cell line 4T1	[61]

Cancer-specific epigenetic mechanisms involved in *FUS1* gene suppression have also been reported. Generally, loss of a gene expression in tumors occurs ~10 times more frequently due to CpG islands hypermethylation of the promoters than due to mutations [62]. Histone H3K9 methylation patterns (H3K9me1-3) are also responsible for gene silencing [63], whereas H3 acetylation leads to the opposite effect [64]. As for *FUS1*, no CpG methylation in the promoter was demonstrated in lung, nasopharyngeal, and breast cancers, even though promoter hypermethylation in the neighboring gene *RASSF1* was detected [65, 66, 47, 10]. However, partial promoter methylation of the *FUS1/TUSC2* gene was reported in head-and-neck [67] as well as in 20% of NSCLC cancers [68]. As for histone modifications, predominance of H3 acetylation (H3K9ac) over methylation (H3K9me3) was reported [66].

**Fig. 2:**
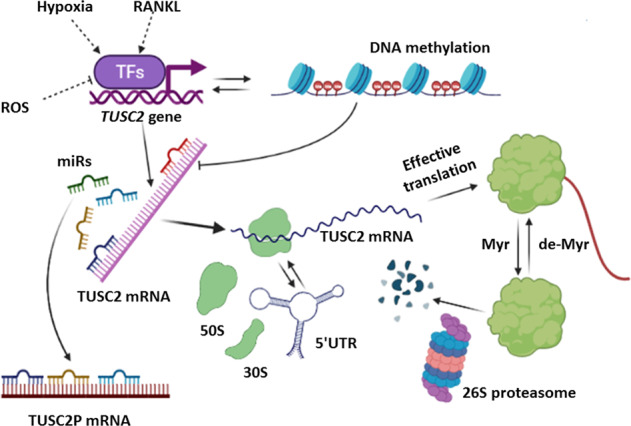
Transcriptional, post-transcriptional, and post-tranlsational regulation of Fus1/Tusc2. Fus1/Tusc2 gene is regulated on transcriptional level by different physiological and pathological factors such as ROS, hypoxia, and differentiation molecules (e.g., RANKL). In some tumors (e.g., NSCLC), DNA methylation may lead to down-regulation of TUSC2 gene transcription. Binding of microRNAs (miRs) to 3’ and 5’ untranslated regions (UTR) of TUSC2 mRNA suppresses its translation. mRNA for TUSC2 pseudogene (TUSC2P) sequesters miRNAs and prevents their binding to TUSC2 mRNA. During translation, 5’ UTR can adopt conformation preferring stoppage of ribosomes along TUSC2 mRNA observed in NSCLC tumor cells. After effective translation, Fus1/Tusc2 undergoes myristoylation (Myr) which is necessary for a protein stability in normal cells; in the absence of Myr tail (in tumor cells), protein is degraded at faster rate via proteasome machinery.

Physiological stimuli affecting *FUS1/TUSC2* mRNA levels vary in their origin. The expression is reported to be downregulated by reactive oxygen species (ROS) [69, 70], and upregulated by hypoxia [58] and the factors stimulating differentiation [71]. Summary of mechanisms regulating Fus1 levels is presented in Fig. 2. However, a fuller understanding of how *FUS1/TUSC2* expression is regulated by physiological and pathological stimuli is still lacking.

## Protein motifs and posttranslational modifications of FUS1/TUSC2

Human FUS1/TUSC2 is a small (110 aa) protein with an estimated MW of 12 kD. The protein is basic and predicted to have a pI (isoelectric point) of 9.69 [10]. According to a computer-based modeling, FUS1/TUSC2 lacks transmembrane domains, is highly hydrophobic and contains helix-coil domain secondary structures [53]. A 3D model for FUS1/TUSC2 protein is shown in Fig. 1B. Tertiary FUS1/TUSC2 structure is still debatable. Below are the functional motifs identified in the protein sequence

1. At the N-terminus, FUS1/TUSC2 contains a myristoylation signal (Met-Gly-X-X-X-Ser/Thr), and experiments confirmed that FUS1 is myristoylated [53] (Fig. 1C).
2. Motif-based profile scanning found a protein kinase A interaction site, an A-kinase anchoring protein interaction site, and a PDZ class II domain [53].
3. *In silico* analysis revealed that protein fragment 54–65 aa is highly homologous to the EF-hand Ca^2+^-binding domains found in calmodulin as well as in mitochondrial proteins MICU1 and LETM1 [24, 25] (Fig. 1C).
4. The N-terminal fragment of FUS1/TUSC2 (positions 45–110) is 53% homologous to myristoyl-binding domain of recoverin, a typical Ca^2+^/myristoyl switch protein. Remakably, 9 of 11 key amino acids that form myristoyl-binding hydrophobic pocket of recoverin share similarity with the FUS1 protein [24, 25] (Fig. 1C).
5. Fus1/Tusc2 of *C. elegans* carries a bipartite NLS (nuclear localization signal) (residues 84–101) and possesses weak similarity to DNA-directed RNA polymerase subunit A [10].
6. The N-terminus of Fus1/Tusc2 displays 40% similarity to the DNA-binding domain of IRF7 [69].
7. The Fus1/Tusc2 with pos. 57–65 is identical to the 9 aa transactivation domains of transcription factors (TFs) p53, NFAT, and NFκB as predicted by 9aa TAD tool [72].

Myristoylation is extremely important for cell localization and functioning of the FUS1/TUSC2 protein [53]. N-myristoylation is a post-translational modification consisting of the removal of the N-terminal methionine from a protein and ligation of the released NH2 group of glycine to the residue of myristic acid, a 14-carbon saturated fatty acid (Fig. 1D) [73]. The resulting lipid tail participates in protein folding and anchoring myristoylated proteins to different membrane compartments, where they execute their functions (Fig. 1E) [74, 73, 75]. A mutant form of FUS1/TUSC2 missing the myristoyl tail has a shorter half-life (6 h *vs* 12 h) due to improper protein folding and increased proteasome degradation [53]. Also, myristoylation-deficient FUS1/TUSC2 loses its characteristic mitochondria/ER localization and its abilities to induce apoptosis and suppress tumor cell proliferation in vitro. Importantly, it also acquires the abilities to promote tumor growth and metastases in vivo [53]. Therefore, loss of myristoylation may be considered a key event that leads to insufficiency of Fus1 function, when compared to mutations and hypermethylation [14, 53].

The predicted Ca^2+^ EF hand binding motif and hydrophobic binding pocket of FUS1/TUSC2 prompted it to be classified as a novel calcium/myristoyl switch protein (Fig. 1D) [24]. Its involvement in Ca^2+^-dependent signaling have been reported for different cell types including CD4^+^ T lymphocytes, kidney epithelial cells, osteoclasts, and mouse embryonic fibroblasts [71, 23, 24, 23, 25]. The presence of myristoylation and Ca^2+^ binding motifs in one protein is pivotal for regulation of cellular processes. In has been established that Ca^2+^/myristoyl switch proteins, in response to Ca^2+^ binding to EF-hand motifs, release their lipid myristoyl tail from a protein hydrophobic pocket and anchor it to membranes during Ca^2+^ elevations (Fig. 1E) [74, 75].

The Fus1/Tusc2 protein can undergo other posttranslational modifications such as arginine mono-methylation (R9) [76], phosphorylation (S50, site for PKA and RSK) [77], and acetylation (K93) [78]. Moreover, NetPhos 3.1 software (http://www.cbs.dtu.dk/services/NetPhos/) predicts phosphorylation at S4 (site for CDC2), S6, T46, and T70 (PKC) (Fig. 1C). Thus, several functional protein motifs and a spectrum of potential posttranslational modifications facilitate Fus1/Tusc2 involvement in sensing and execution of various cellular programs.

## FUS1/TUSC2 in mitochondrial Ca^2+^ regulation

### Mitochondrial Ca^2+^ transport: main players

**Fig. 3:**
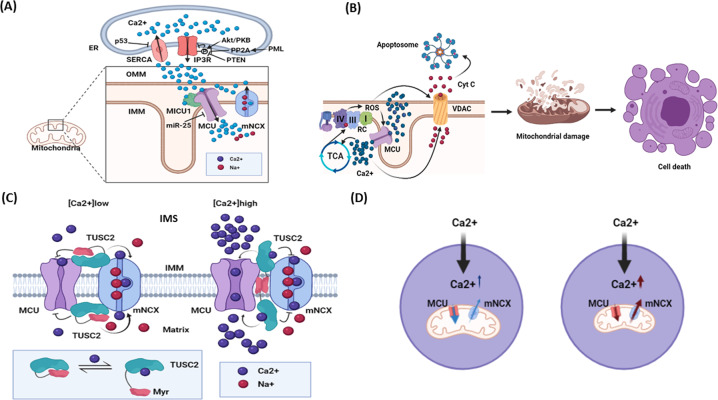
Role of intracellular Ca^2+^ signaling in cell decisions and potential place of Fus1/Tusc2 in Ca^2+^ signaling network. **A** Mitochondrial calcium transport at the endoplasmic reticulum (ER)/mitochondria interface and its regulation by tumor suppressors and protooncogenes. Ca^2+^ released from ER via inositol triphosphate receptors (IP3Rs) is taken up by mitochondria via Ca^2+^ uniporter (MCU). MICU1 stimulates opening of the MCU channel after Ca^2+^ binding to its EF-hand motif and fine-tunes Ca^2+^ currents. Ca^2+^ accumulated in mitochondria can be transported back into inter-organellar space by the mitochondrial Na^+^/Ca^2+^ exchanger (mNCX) and captured by Ca^2+^ ATPases in ER (SERCA). IP3Rs activity is negatively regulated by pro-survival/pro-tumorigenic Akt-mediated phosphorylation. This effect is counteracted by tumor suppressors: [1] PML which binds to IP3R and [2] PTEN which hydrolyzes phosphoinositides necessary for Akt/PKB activation. p53 inhibits SERCA activity, which leads to cytosolic Ca^2+^ retention potentially promoting senescence and cell death. Another pro-survival mediator, miR-25, down-regulates MCU and reduces cell susceptibility to apoptosis. **B** Cell death induced by Ca^2+^ overload. Massive Ca^2+^ uptake by mitochondria results in overstimulation of the tricarboxylic acid (Krebs) cycle (TCA) supplying the respiratory chain (RC) with redox components. This process induces reactive oxygen species (ROS) overproduction leading to a redox modification of MCU that intensifies Ca^2+^ uptake, therefore forming a positive feedback loop. Accumulation of Ca^2+^ along with enhanced ROS production triggers opening of the mitochondrial permeability transition pore and release of cytochrome C via voltage-dependent anion channel (VDAC). Mitochondrial disruption and interaction of cytochrome C with Apaf-1 leads to apoptosome formation triggering cell death. **C** Proposed mechanism of action of Fus1/Tusc2 protein. At low Ca^2+^, Fus1/Tusc2 maintains its inactive state by adopting a conformation with the myristoyl tail hidden inside of the hydrophobic pocket. This conformation keeps mtCU inactive and mNCX active, thus preventing Ca^2+^ rise in mitochondrial matrix. When Ca^2+^ is elevated in the intermembrane space (IMS) or matrix, Fus1/Tusc2 releases its lipid tail that anchors to the inner mitochondrial membrane (IMM), thus maintaining the MCU at the open state and preventing mNCX activation. Overall, these actions help to increase Ca^2+^ in mitochondrial matrix to the levels necessary for TCA stimulation. **D** A schematic diagram demonstrating how deficiency in mitochondrial Ca^2+^ accumulation affects cytosolic Ca^2+^. At the steady state, adequate and coordinated mtCU and mNCX activities allow Ca^2+^ to accumulate in mitochondria and maintain cytosolic Ca^2+^ at the moderate levels. However, inhibition/loss of mtCU and activation of mNCX would result in cytosolic Ca^2+^ retention and alterations in the pattern of activation of Ca^2+^-dependent proteins.

MitoCa^2+^ transport is balanced by Ca^2+^ import and export. The main route for mitoCa^2+^ entry is mitochondrial Ca^2+^ uniporter holocomplex (MCU_cx_), consisting of membrane channel protein MCU (*m*itochondrial *C*a^2+^ *u*niporter) and MCU regulatory proteins MICU1-3, MCUb, MCUR1, EMRE [79–81]. MCU is an inner mitochondrial membrane protein that forms a pentameric channel with a selectivity filter for Ca^2+^ ions (a DIME motif) (Fig. 3A) [82, 83]. MCU becomes permeable for Ca^2+^ when ion concentration reaches 1-10 μM, which is only possible at the contact with the endoplasmic reticulum (ER) where Ca^2+^ is released *via* inositol-1,4,5-triphosphate receptors (IP3Rs). The 1-10 μM Ca^2+^ threshold is set by the Ca^2+^-binding EF-hand motif of the mitochondrial protein MICU1 (*mi*tochondrial *C*a^2+^ *u*ptake*1*) (Fig. 3A) [84, 85]. In the absence of MICU1, MCU transports Ca^2+^ at much lower cytoplasmic concentrations (hundreds of nM range) accompanied by mitoCa^2+^ overload [80]. However, other reports suggested that the lack of MICU1 inactivates MCU in a Ca^2+^-dependent manner at a faster rate *via* autoinhibition. Accordingly, MICU1 plays a gatekeeper role for MCU and prevents its premature inactivation [86]. MICU1 heterodimerizes with its analogs, MICU2 and MICU3. Thus, MICU1/MICU2 heterodimer fine-tunes Ca^2+^ currents: MICU1 stimulates Ca^2+^ uptake at high cytosolic Ca^2+^ level while MICU2 inhibits MCU at low cytosolic Ca^2+^ content [87].

The importance of MCU in tumor growth is based on its involvement in cell death. Malignant cells benefit from reduction of mitoCa^2+^ uptake. This is especially important as tumor cells experience increased ROS production. Interestingly, MCU contains ROS sensing cysteines in its N-terminus. Their oxidation promotes MCU_cx_ assembly, persistent channel activity, and mitoCa^2+^ overload following by opening the permeability transition pore, which triggers cell death (Fig. 3B) [80]. However, for some breast cancers it was reported that activation of MCU promotes cancer cells motility, invasion, and growth [79]. So, it is not surprising that mitoCa^2+^ accumulation undergoes regulation by tumor suppressors. Thus, p53 directly interacts with the Ca^2+^-ATPase in ER, promotes its negative oxidative modification, and stimulates enhanced Ca^2+^ release from ER with further Ca^2+^ transfer to mitochondria *via* the MCU protein (Fig. 3A) [88]. The lipid/protein phosphatase PTEN binds IP3R and, thus, counteracts PKB/Akt phosphorylating and inhibiting IP3R; this results in a higher mitochondrial Ca^2+^ accumulation and apoptosis (Fig. 3A) [89]. On the other hand, down-regulation of the MCU mRNA by miR-25 is accompanied by increased cancer cell survival and apoptosis resistance (Fig. 3A) [90].

Extrusion of Ca^2+^ from mitochondria into cytosol is mediated by NCLX (Na^+^/Ca^2+^/Li^+^ exchanger), a mitochondrial form of Na^+^/Ca^2+^ exchanger (mNCX), and Ca^2+^-binding EF-hand motif containing LETM1, a 2H^+^/Ca^2+^ exchanger (Fig. 3A) [79, 91]. Diminished NCLX activity leads to mitoCa^2+^ overload and elevated ROS production due to the activation of Krebs cycle [92]. In a few tested non-tumor cell models, these events led to cell death. In cancer cells, however, although increased ROS blocked cell proliferation, they also induced metastasis *via* the ROS/HIF1α signaling axis [93]. Therefore, Ca^2+^ transients in mitochondria play a decisive role in the normal and tumor cell biology.

### Potential mechanism of mitochondrial Ca^2+^ regulation by Fus1/Tusc2 protein

Localization of Fus1/Tusc2 in mitochondria and the discovery of a Ca^2+^-binding domain in its structure [22, 24] suggested that Fus1/Tusc2 could regulate mitoCa^2+^ accumulation. Indeed, Fus1 loss reduced mitoCa^2+^ accumulation resulting in retention of Ca^2+^ in cytosol [24, 23]. Like MICU1, Fus1 showed a dual effect on mitoCa^2+^. Fus1-deficient cells had increased steady-state mitoCa^2+^ levels, while decreased cytoCa^2+^ levels. Moreover, during Ca^2+^ response, Fus1^−/−^ mitochondria accumulated Ca^2+^ at a faster initial rate than WT mitochondria and reached higher Ca^2+^ values at the peak of response. However, levels of mitoCa^2+^ declined faster during the recovery phase of Ca^2+^ response in Fus1-deficient cells [24, 23, 25]. This effect of Fus1/Tusc2 deficiency on mitoCa^2+^ was partially alleviated by inhibition of mNCX responsible for mitoCa^2+^ extrusion [24]. Accordingly, Fus1/Tusc2 may set a threshold for Ca^2+^ uptake similar to MICU1 and prevent mitoCa^2+^ accumulation at steady-state or rapid Ca^2+^ accumulation after initial rise in cytoCa^2+^ (Fig. 3C, left). However, when cytoCa^2+^ concentration reaches high values, we propose that Fus1/Tusc2 binds Ca^2+^ ions, and releases its lipid tail that anchors protein to the mitochondrial membrane, thus maintaining mtoCa^2+^ uptake. Finally, Fus1/Tusc2 inhibits mNCX and promotes maximal Ca^2+^ accumulation in mitochondria (Fig. 3C, right) [24, 23, 25]. When levels of cytoCa^2+^ start recovering, drop in Ca^2+^ initiates reversed events: Ca^2+^ import *via* MCU declines and mNCX accelerates Ca^2+^ export out of mitochondria. Although regulation of cytoCa^2+^ by mitochondria is complex, usually deficiency in MCU translates into cytoCa^2+^ retention (Fig. 3D) [94]. Sustained cytoCa^2+^ results in prominent activation of Ca^2+^-dependent proteins, i.e., NADPH oxidase [95], CAMKII [94] or Miro1 [80].

**Fig. 4:**
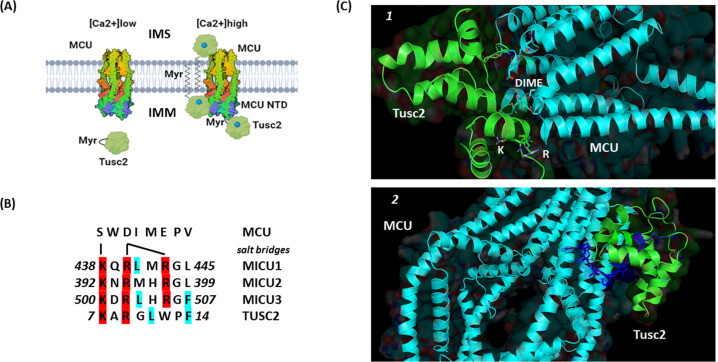
Hypothetical mechanisms of Fus1/Tusc2 and MCU (mitochondrial Ca^2+^ uniporter) interaction. **A** A proposed mechanistic model of inter-protein interaction between Fus1/Tusc2 and MCU. At low Ca^2+^ levels, Fus1/Tusc2 maintains a closed conformation with its myristoyl tail hidden in a hydrophobic pocket that promotes protein folding, stabilizes inactive state, and prevents premature protein degradation. At high Ca^2+^ levels, myristoyl tail is released triggered by Ca^2+^ binding to EF-hand of Fus1/Tusc2. Two possible scenarios could be proposed after the tail release: [1] the lipid tail anchors Fus1/Tusc2 to the membrane followed by the Fus1/MCU interaction in the mitochondrial intermembrane space (IMS) or matrix side of the inner membrane (IMM), or [2] the lipid tail of Fus1/Tusc2 binds the hydrophobic surface of the MCU N-terminal domain (NTD) and affects activity/kinetics of the mitochondrial channel. **B** The Fus1/Tusc2 protein motif, which may interact with the MCU DIME motif (Ca^2+^ selectivity filter). The 7-15 aa fragment from Fus1/Tusc2 was compared with motifs of the MICU1-3 proteins responsible for binding to MCU in the IMS. Critical amino acids responsible for formation of salt bridges with the DIME motif in MCU are shown in red. Blue/green color highlights identical residues in similar positions in Fus1/Tusc2 as compared to MCU1-3 proteins. **C** Docking simulation of predicted interaction between the MCU/EMRE complex and Fus1/Tusc2 protein. In the configuration *1*, Fus1/Tusc2 interacts with MCU close to its DIME sequence (Ca^2+^ selectivity filter) in the mouth of the channel. Lys (K) and Arg (R) from the Fus1/Tusc2 motif homologous to MCU-binding motif of MICU1-3 (KxRxxRGx) (see Fig. 4B) are positioned against the DIME motif. Critical Ser and Asp residues in the DIME motif required for formation of salt bridges as well as potential Fus1/Tusc2-binding sequence (including K and R) are marked with licorice sticks. (see 3B and text). In the configuration *2*, Fus1/Tusc2 interacts with the MCU/EMRE complex via the 81-96 aa fragment (highlighted in blue) that was experimentally shown to be involved in the inter-protein interactions. Docking simulation has been performed using the ClusPro server [143] and visualized using PyMOL software (The PyMOL Molecular Graphics System, Version 2.0 Schrödinger, LLC; https://pymol.org/2/#page-top). The MCU sequence was retrieved from the Protein Data Bank (acc # 6O5B).

Initially, it was proposed that MICU1 regulates MCU-mediated Ca^2+^ currents *via* Ca^2+^/myristoyl switch mechanism [96]. However, the MICU1 and MCU interaction is mediated *via* direct protein binding. At the MICU1 C-terminus, KQRLMRGL peptide represents an MCU-binding domain interacting with with DIME motif *via* salt bridges [97]. Structural analysis of the MCU N-terminal domain (NTD) surprisingly revealed unidentified lipid molecule bound to a hydrophobic protein surface formed by residues in the L1 loop, two helices (α2 and α3) and C-terminal tail. This lipid was described as a linear lipid-like structure consisting of 13–16 carbon atoms that is similar to a tetraethylene glycol molecule [67]. It is noteworthy that myristic acid (a substrate for N-terminal myristoylation) is a saturated linear long-chain lipid with a 14-carbon backbone complying with the features of an unidentified lipid. Therefore, Fus1/Tusc2 could regulate the activity of MCU *via* a novel mechanism of inter-protein lipid tail exchange. We suggest that the myristic acid residue of FUS1/TUSC2 protein released from the hydrophobic pocket after Ca^2+^ binding to EF-hands could interact with the hydrophobic surface of MCU and maintain its Ca^2+^ transporting function (Fig. 4A). This is likely in view of the fact that Fus1/Tusc2 has the amino acid sequence KARGLWPF resembling MCU-binding domain of MICU1–3 proteins (Figs.1, 4B and C). Moreover, FUS1/TUSC2 can potentially interact with MCU *via* its inter-protein interaction site at the C-terminus (positions 81-96) (Figs. 3 and 4C). This mechanism needs further elucidation by functional and structural studies.

### Putative Fus1 protein-protein interaction *via* myristoyl tail exchange

An intriguing opportunity for protein-protein interactions was demonstrated by mutational analysis of FUS1/TUSC2 binding to a pro-tumorigenic tyrosine kinase c-Abl [98, 99]. Initiated by discovery of a truncated form of FUS1/TUSC2 (deletion of C-terminal 83-110 aa) in tumor cells, Lin et al. showed that the FUS1/TUSC2 81-96 aa C-terminal peptide linked with stearate inhibits c-Abl activity and facilitates its degradation. On the contrary, the FUS1/TUSC2 N-terminal peptide 1-80 aa, although capable of binding to c-Abl, was unable to inhibit its kinase activity [98]. Like FUS1/TUSC2, c-Abl is a myristoylated protein. It is activated by releasing the myristoyl tail from the hydrophobic pocket and conforming to an active open state, which allows c-Abl to bind its substrates *via* the SH2-domain [100]. FUS1/TUSC2 mutational analysis showed that while the N-portion of FUS1/TUSC2 binds to myristoyl-binding pocket of c-Abl, the C-terminal portion of FUS1/TUSC2 (containing c-Abl inhibitory peptide 81-96 aa) interacts with the ATP-binding site in the N-lobe of c-Abl. Alternatively, FUS1/TUSC2 could interact with an activation loop in the C-lobe of c-Abl [99].

Initially, it was thought that myristoylation is exclusive for targeting proteins to certain membrane compartments but, nowadays, this posttranslational modification is appreciated to play roles in protein folding and prevention from premature degradation [100, 101, 53]. A synthetic ligand for myristoyl binding pocket named GNF-5 maintains closed inactive conformation of c-Abl apo form or convert open conformation to a closed one [102]. Therefore, one could speculate that the myristoyl tail of FUS1/TUSC2 inserted into the hydrophobic pocket of c-Abl inhibits its tyrosine kinase activity. This tail-pocket interaction can be an important feedback loop in the network of interactions initiated by RTKs. Thus, platelet-derived growth factor receptor (PDGFR) stimulates signaling molecules such as PLCγ1, c-Src, and c-Abl. In turn, PLCγ1 generates IP3 and promotes Ca^2+^ release from the ER [103].

We suggest that activation of FUS1/TUSC2 by Ca^2+^ leading to the release of its myristoyl tail could suppress c-Abl activity. Noteworthy, c-Abl-deficient B cells exhibited reduced Ca^2+^ flux in response to antigen receptor or CD19 stimulation [104] reinforcing the concept of mutual regulatory loops between FUS1/TUSC2 and c-Abl.

### Ca^2+^ signaling fine-tuned *via* Fus1/Tusc2 impacts cell fate

Maintaining mitoCa^2+^ at moderate levels is important due to the effect of Ca^2+^ on ROS production and cell death enhancing effect of Ca^2+^ overloading [53, 77]. The MICU1-3 proteins fine-tune MCU activity by setting a threshold to filter out mitoCa^2+^ elevations at low cytosolic Ca^2+^ levels (<350 nM) and cooperatively increasing MCU currents at high Ca^2+^ elevations (Fig. 3A) [89, 97]. Similarly, FUS1/TUSC2 may controls basal mitoCa^2+^ by inactivating MCU_cx_, which prevents rapid initial Ca^2+^ accumulation and promotes adequate mitoCa^2+^ elevation (Fig. 3C) [23, 24, 23, 25]. This translates into appropriate activation of Krebs cycle and sufficient formation of antioxidants (NADH, NADPH) maintaining ROS at low level. Accordingly, loss of antioxidant defense in *Fus1*-deficient cells [24, 25, 49, 69], would reduce cell proliferation, tissue repair, and promote cell death. Indeed, MICU1 deficiency accompanies compromised liver regeneration after partial hepatectomy due to inflammation, overloaded mitoCa^2+^, blocked proliferation, and increased necrosis of hepatocytes [105].

Compromised capability of *Fus1*^−/−^ adult stem cells to repopulate tissues was reported in aged *Fus1*^−/−^ animals (e.g., hair follicles, thymus) [23] or younger mice after exposure to radiation (e.g., GI crypt epithelial cells, melanocyte stem cells) [49]. In vitro data suggest that Fus1 is involved in bone remodeling shaped by bone deposition (osteoblasts) and resorption (osteoclasts) [106]. Silencing of *Fus1/Tusc2* gene in bone marrow precursor cells in vitro diminished RANKL-induced osteoclast differentiation without affecting osteoblast formation [71]. Thus, it is possible that *Fus1/Tusc2* loss in vivo could also affect bone remodeling, shifting it towards bone deposition that would reduce tissue repair potential [106] corroborating with accelerated aging and development of age-related disorders in *Fus1*^−/−^ mice [23].

Thresholds in the mitogenic signal transduction demand tight control of activation of proteins involved in cell proliferation. Malfunction of key tumor suppressors (e.g., E3 ligase PML) may switch cell fate from senescence to malignant transformation [50, 107]. The ability of Fus1/Tusc2 to calibrate Ca^2+^ responses would translate into adequate cell responses based on the characteristics of input signals such as signal strength. In this regard, Fus1/Tusc2 reminds other tumor suppressors (e.g. PTEN, Spry), which prevent cells from over-stimulation by mitogenic signals maintaining their survival and responsiveness to proliferation signals [108–112].

## Regulation of apoptosis by FUS1/TUSC2

Numerous studies demonstrated that *FUS1/TUSC2* overexpression in cancer cells that lack 3p21.3 or *FUS1/TUSC2* gene/expression induces cell death [2, 46, 47, 53, 113, 114]. Co-expression of *FUS1/TUSC2* and *p53* synergistically increased apoptosis in NSCLC cells. This synergistic effect was associated with the ability of Fus1/Tusc2 to down-regulate expression of MDM2, an E3 ubiquitin ligase that suppresses p53, and thereby stabilizes p53 levels. Importantly, combined effect of Fus1 and p53 co-expression required Apaf-1 that mediates mitochondria- and caspase3-dependent apoptosis^(113)^. Additionally, in thyroid cancer cell lines, overexpression of *FUS1/TUSC2* increased levels of Smac/Diablo that blocks caspase regulatory inhibitors of apoptosis proteins and cytochrome *c* [115]. Protein Chip array and SELDI-TOF mass spectrometry revealed direct interaction between PDZ domains of FUS1/TUSC2 and Apaf [116] corroborating the link of FUS1 with the Apaf-1-mediated apoptosis.

## Fus1/Tusc2 in cellular senescence

### Cellular senescence: main players

**Fig. 5:**
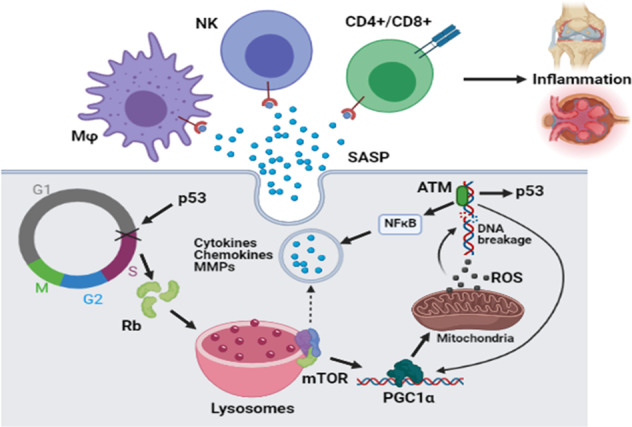
Senescence-associated secretory phenotype (SASP). G1/S cell cycle block (marked by a cross) driven by p53 is accompanied by accumulation of Rb protein, a critical suppressor of G1/S transition. Rb-triggered up-regulation of lysosomal compartment stimulates mTOR. In turn, mTOR activates PGC1α, a transcription factor involved in mitochondrial biogenesis. Up-regulated mitochondrial compartment accompanied by increase in respiration leads to increased ROS formation. It is followed by DNA damage, a main trigger of the ATM-mediated activation of NFκB pathway, p53, and PGC1α. As a result, positive feedback loops are forming that lead to senescence. NFκB-regulated transcription program triggers elevation of cytokines, chemokines, metalloproteinases (MMPs), and other molecules secreted by senescent cells (senescence-associated secretory phenotype, SASP). The secreted molecules attract immune cells, which induce inflammation in tissues populated with senescent cells.

Cellular senescence refers to cellular aging characterized by a highly stable cell cycle arrest accompanied by biochemical and morphological alterations [117, 118]. The phenomenon of a limited number of cell divisions discovered by Leonard Hayflick in 1961 became known as the Hayflick limit [119]. A role of p53 in senescence is evident from its effect on cell cycle arrest usually mediated by up-regulation of p21, an inhibitor of cyclin-dependent kinases CDK1, CDK2, and CDK4/6 required for the G1/S transition [120, 121]. Senescence can be induced by oncogene overactivation, oxidative stress, genotoxic drugs, radiation, CDK suppression, demethylating and acetylating agents, etc. [121]. Cell cycle arrest accumulates unphosphorylated form of Rb protein, upregulating the lysosomal compartment and subsequently stimulating mTOR signaling (Fig. 5) [122]. mTOR signaling, in turn, up-regulates mitochondrial biogenesis *via* TF PGC1α. Increased mass and dysfunctional state of mitochondria enhance intracellular ROS and DNA damage. Downstream of this cascade, DNA damage kinases (i.e., ATM) further activate the AKT/mTOR signaling axis stimulating PGC1α and creating a positive feedback loop stabilizing senescent state (Fig. 5) [117, 118].

DNA damage is responsible for activation of the NFκB pathway regulating senescence-associated secretory phenotype (SASP); senescent cells secrete proinflammatory cytokines (IL-1β, IL-8, IL-6), chemokines (MCP-1, CCL3, CXCL1), matrix metalloproteinases (MMP3, MMP9), etc. (Fig. 5) [123, 117, 118]. Moreover, SASP reinforces senescence *via* autocrine pathway and induces it in neighboring cells. Secreted cytokines and chemokines attract immune cells necessary for clearance of senescent cells (Fig. 5). However, accumulation of senescent cells results in the development of chronic inflammatory disorders [124, 125, 121]. Initially considered an in vitro phenomenon, senescence was recently confirmed in vivo; transplantation of senescent cells from ear cartilage into knee joint caused an osteoarthritis-like phenotype in mice [126]. Also, senescent fibroblasts can promote growth and proliferation of tumor cells *via* secretion of SASP intermediates (e.g., fibroblast growth factors 10 and 19, IL-1β), epithelial-mesenchymal transition (IL6, MMP2-3), and immune evasion mechanisms (i.e., IL-6 drives accumulation of suppressive myeloid cells and their activity) [124]. Therefore, senescence and SASP-mediated chronic inflammation could underlie the development of aging-related diseases including tumors.

### Ca^2+^ signaling and senescence

Fine-tuned control of cellular Ca^2+^ signaling by tumor suppressors and oncogenes [127] established that increased cellular Ca^2+^ (cytosolic and/or mitochondrial) results in senescence or apoptosis, whereas moderate levels of Ca^2+^ favor cell proliferation. Ca^2+^ regulates SASP via activation of calpain that converts pro-IL-1α into functional IL-1α^(128)^. Another Ca^2+^ effector is NFAT, a TF regulated by calmodulin/calcineurin complex that has both pro-proliferative and pro-senescent effects. In particular, NFAT induces p53 by suppressing expression of ATF3, a p53 negative regulator. NFAT acts as a pro-senescent factor by activating expression of IP3R2 that mobilizes Ca^2+^ from ER elevating cytosolic and mitoCa^2+^ levels [128].

IP3Rs are instrumental in cancer cells, where the number of oncogenes and tumor suppressors cross their paths [127]. Pro-survival/pro-tumorigenic PKB/Akt phosphorylates and inactivates IP3R3. Tumor suppressor protein PML binds to IP3R3 and recruits protein phosphatase PP2A to dephosphorylate IP3R3 leading to IP3R3-marked opening (Fig. 3A). Consequently, Ca^2+^ accumulation in mitochondria would increase ROS production leading to a senescent state (Fig. 3B). It is noteworthy that abrogation of *MCU* helps cells avoid senescence [128] and apoptosis, and promotes uncontrolled proliferation, migration, and metastases [80].

### Fus1/Tusc2-dependent processes mediating cellular senescence

#### Proliferation

The role of Fus1/Tusc2 in the control of senescence appears to be a part of its role as a TSG. *FUS1/TUSC2* overexpression in tumor cells is associated with cell cycle arrest at G1/S [47] and G2/M [115] transition checkpoints. Deletion or silencing of *Fus1/Tusc2* accelerates proliferation of activated mouse CD4^+^ T cells [24] and human tumor cells [129]. Upregulation of miR-197 miRNA that increases *FUS1/TUSC2* levels and, thus, suppresses tumor metastasis was attributed to the ability of FUS1 to regulate proliferation of human glioblastoma cells [129].

#### Genotoxic stress

In several models of cell injury, *Fus1*^−/−^ cells showed increased levels of genotoxic stress and senescence markers compared to wild-type counterparts. For example, after intraperitoneal injection of asbestos, infiltrating cells from Fus1^−/−^ mice had higher levels of γH2AX, DNA damage response molecule, and phosphorylated pro-inflammatory NFκB and ERK1/2. This was associated with increased number of macrophages and accelerated accumulation of granulocytes in the peritoneal cavity. Among others, IL-1β, a signature cytokine of SASP, was up-regulated in peritoneal Fus1^−/−^ cells.

The increased sensitivity to irradiation in Fus1^−/−^ mice was accompanied by accelerated cell cycle arrest, aberrant mitosis, lack of proper DNA repair (mitotic catastrophe), early activation of p53, and death of gastrointestinal crypt cells, which are especially susceptible to ionizing radiation [49]. Thereby, Fus1-deficient mice demonstrated enhanced cell damage upon challenging stimuli like other TSG deficiency models (i.e., p27) [8].

#### Clearance of senescent cells

The most striking alteration in the chemokines/cytokines profile of *Fus1*-deficient mice was down-regulation of RANTES/CCL5, a chemokine for T and NK cells [70]. Since T and NK cells are necessary for clearance of senescent cells [130], lack of CCL5 production in *Fus1*^*−*^^*/−*^ mice could lead to accumulation of senescent cells during the course of lifetime and result in chronic inflammation, aging-related diseases, and malignant transformation.

## Consequences of Fus1/Tusc2 deficiency at organismal level

### Fus1/Tusc2-associated systemic pathologies

Several Fus1-dependent pathologies developing in mice have been reported following the targeted *Fus1* inactivation. These could be divided into two groups:

#### Spontaneous pathologies developed in young or middle-aged mice and progressing with time

- Chronic systemic inflammation [23].
- Progressive development of SLE-like autoimmune disease in some mice (incomplete penetrance) (vasculitis, glomerulonephritis, anemia, circulating autoantibodies) [22].
- Increased frequency of spontaneous vascular tumors [22].
- Preponderance of aging signs that include lordokyphosis, absence of vigor, diminished hair regrowth, reduced sperm count and motility, enlarged seminal vesicles, and compromised stem cells self-renewal [22, 23].
- Early development of aging-associated diseases:
  1. Premature progressive hearing loss (higher threshold for sound intensity, longer latency to respond to sound, and smaller amplitude in auditory brainstem responses (ABR) waves, compared to wild-type mice [50]. The hearing loss corresponded with impaired PTEN/Akt/mTOR pathways in cochlear cells and was ameliorated by administration of N-acetyl cysteine, an antioxidant agent [50].
  2. Impairments in olfactory and spatial memory at a relatively young age (4-5 months old), as indicated by habituation test, hidden cookie test, and Morris water-maze test [51].

#### Injury-induced pathologies in young mice

- Higher sensitivity to γ-irradiation [48, 49].
- Higher sensitivity to peritoneal asbestos injury [70].
- Resistance to *A. baumanii* lung infection [21].

Most likely, defects in common Fus1-dependent mechanism(s)/pathways that are discussed below underlie these pathologies.

### The mTOR pathway activation

*Fus1*^−/−^ mice showed prominent mTOR signaling activation and oxidative stress, signature hallmarks of senescence and early aging. In young *Fus1*^−/−^ mice, cochlear cells are distinguished with reduction of antioxidant enzymes (mitochondrial SOD2, PRDX1) and concomitant activation of the Akt/mTOR pathway (decrease in PTEN levels, up-regulation of phospho-Akt and S6). This molecular pattern was associated with low-grade chronic inflammation observed in bone marrow cells in the temporal bone surrounding the cochlea [50]. During senescence, activation of Akt/mTOR (for example, *via* DNA damage/ATM pathway) stimulates PGC1α followed by up-regulation in mitochondrial biogenesis. Mitochondria-derived ROS damage DNA and, thereby, maintain mTOR activation. Additionally, mTOR regulates translation of mRNA related to SASP *via* MAP kinase-activated protein kinase 2 phosphorylating protein ZFP36L1 responsible for mRNA degradation ^(117)^. Therefore, it is not surprising that chronic inflammation accompanies senescent phenotype of cochlear *Fus1*^−/−^ cells [50].

### Oxidative stress

Increased ROS stabilize p21, a p53 activator and key suppressor of cell proliferation in senescence, and inhibit autophagy 23. Increased ROS production was evident in *FUS1/TUSC2*-deficient head-and-neck cancer cells JHU012 [70] and in mouse splenocytes [24]. Additionally, cochlear cells from *Fus1*^−/−^ mice demonstrated up-regulation of antioxidant defense proteins (Prdx1) in steady-state epithelial cells as well as gradual decrease in the expression of Sod2 and Prdx1 [50]. Further, *Fus1*^−/−^ primary mouse embryonic fibroblasts and immortalized kidney epithelial cells showed defects in respiration such as significantly decreased maximal mitochondrial respiration and respiratory reserve capacity, likely due to down-regulation of mitochondrial respiratory proteins [127]. However, upon challenging conditions (i.e., irradiation) *Fus1*-deficient cells displayed delayed up-regulation of Sod2, a mitochondrial form of antioxidant superoxide dismutase [49]. Treatment with antioxidants (Tempol, pyroxidamine) restored Sod2 expression and significantly improved survival of whole-body irradiated Fus1-deficient mice [48]. Another antioxidant, N-acetylcysteine, rescued expression of Prdx1 and respiratory chain compounds, restored normal mitochondrial morphology, and prevented progression of hearing loss in *Fus1*^−/−^ animals [50].

### Disruption of mitochondrial Ca^2+^ homeostasis

Increased ROS production in *Fus1/Tusc2*-deficient tissues may derive from Fus1 ability to regulate mitoCa^2+^ transport [23–25]. Indeed, inhibition of MICU1 *via* Akt/PKB phosphorylation increased ROS production and downstream Akt/PKB activation [85, 90]. The deficiency in mitoCa^2+^ leads to elevated cytosolic Ca^2+^ and activation of Ca^2+^-dependent proteins in the cytosol. For example, Ca^2+^-dependent stimulation of Miro1 results in the remodeling of long filamentous mitochondria into globe-shaped mitochondria, step prerequisite for autophagosomal degradation [80]. Indeed, presence of globule-shaped giant mitochondria in *Fus1*-deficient epithelial and cochlear cells point to preferred mitochondrial fission in these cells [24, 51]. This process should activate mitophagy, but in *Fus1*^−/−^ mice it rather leads to accumulation of nonfunctional mitochondria. *Fus1*^−/−^ cochlear cells displayed a major down-regulation in the expression of PTEN-induced kinase-1 (PINK1) [51], a sensor of mitochondrial quality control, which directs dysfunctional mitochondria towards autophagy/mitophagy [131]. Importantly, PINK1 protects cells from oxidative stress and premature senescence [132]. Therefore, *Fus1*-deficient cells accumulate dysfunctional mitochondria leading to further increase in ROS production and senescence [50].

### Inflammation

Transcriptomic analysis of *Fus1*^−/−^ CD4^+^ T cells revealed that at the basal level T lymphocytes significantly up-regulate gene expression of secretory pro-inflammatory markers such as *Mmp8-9*, *S100a8-9*, *Lcn2*, *Ltf*, *Retnlg* [24]. Some of these markers (S100a8-9, MMP8-9) are signatures for SASP [133–135] and associate with chronic inflammation observed in *Fus1*-deficient mice [23, 50, 70]. Persistent oxidative stress leads to chronic inflammation, whereas senescence limits stem cell turnover culminating in early aging. Therefore, *Fus1/Tusc2* loss links systemic aging to early senescence, geriatric diseases as well as to tumor escape from immunosurveillance [136].

## Conclusions and clinical relevance

The Fus1-mediated cellular homeostasis is at the crux of its tumor suppressor, anti-inflammatory, and anti-aging activities. Developing *FUS1*-based genetic therapies for cancer patients became an apparent step after revealing its TSG properties.

In preclinical studies, intra-tumoral injection of cationic liposome nanoparticles complexed with plasmid DNA encoding *FUS1/TUSC2* gene (REQORSA or quaratasugene ozeplasmid formely known as Oncoprex) significantly inhibited growth of human NSCLC cells H1299 and A549 in subcutaneously inoculated mice. Administered intravenously, REQORSA suppressed metastases and extended survival of tumor-bearing mice [114]. Phase-I clinical trial reported the effective dosage and safety of REQORSA[13].

Conventional therapy for metastatic NSCLC currently uses epidermal growth factor receptor (EGFR) tyrosine kinase inhibitors such as erlotinib, gefitinib, and osimertinib [137]. Beside toxicity, this type of therapy often leads to treatment-resistant cancer as a result of selecting tumor cell clones carrying mutations. Thus, continuous treatment with erlotinib prescribed to NSCLC patients with overexpression or mutations in EGFR (deletion in 19th exon or L858R point mutation), leads to accumulation of cell clones with T790M mutation in EGFR or up-regulation in other growth receptor signaling pathways (e.g., HGFR, RBB3/PI3K) leading to drug resistance. Osimertinib allows to overcome this issue as it can block EGFR mutated at T790M site; however, resistance to this treatment can also develop after losing tumor cells with T790M [137]. Therefore, combination of EGFR inhibitors with other treatments affecting upstream signaling pathways involved into drug resistance is highly desirable. Ability of Fus1/Tusc2 to suppress receptor and non-receptor kinases as well as sensitize NSCLS cells to chemotherapeutics [14, 98, 138, 139] makes this tumor suppressor a promising therapeutic candidate for combinatorial therapy. Currently, two clinical trials based on combination of Fus1-gene based drug REQORSA and EGFR inhibitors erlotinib (https://clinicaltrials.gov/ct2/show/NCT01455389) and osimertinib (Acclaim-1, https://clinicaltrials.gov/ct2/show/NCT04486833) are underway for treatment of NSCLC patients.

Ongoing revolution in immunotherapy started with a clinical introduction of checkpoint inhibitors significantly improved therapeutic outcomes in patients with different types of cancer (melanoma, breast cancer, colon cancer, Hodgkin lymphoma, etc.) [15–17]. However, effectiveness of immunotherapeutics is limited due to evasion of tumor cells from immunosurveillance mechanisms (e.g., up-regulation of TIM-3 immune suppressive molecules on cancer cells after PD-1 blocking therapy) eventually leading to tumor resistance similar to chemotherapy [18, 19]. Thus, treatments with drugs targeting pathways upstream of drug resistance would be beneficial.

Overexpression of *FUS1/TUSC2* down-regulates mTOR signaling, which stimulates PD-L1, an immunosuppressive ligand up-regulated in many types of tumors including NSCLC. *FUS1/TUSC2*-induced decrease of PD-L1 expression in response to its main inducer, IFN gamma, modifies tumor microenvironment, unleashes T and NK cells from inhibition, and allows effective use of PD-1 blockers [24, 140]. REQORSA and anti-PD-1 combined therapy demonstrated significantly stronger immune response than individual therapies. This resulted in the development of favored anti-tumor response (Th1 differentiation, NK and CD8 + CTL recruitment) and down-regulation of immune suppression signatures (PD-1, CTLA-4, TIM-3) [20]. Recently, a clinical trial Acclaim-2 using REQORSA (also called GPX-001) combined with PD-1 blocking Abs (Pembrolizumab) in treated non-small lung cancer patients has been launched [https://clinicaltrials.gov/ct2/show/NCT05062980].

Thus, studies initiated about 40 years ago to dissect chromosomal aberrations in lung cancer cells culminated in a new promising gene therapy aimed to battle most aggressive lung cancer stages. In addition, Fus1/Tusc2-mediated anti-inflammatory and anti-aging activities provide avenues for development of new approaches to fight conditions of chronic inflammation, infections, premature aging and geriartic diseases.
